# Cupreines and cupreidines: an established class of bifunctional cinchona organocatalysts

**DOI:** 10.3762/bjoc.12.46

**Published:** 2016-03-07

**Authors:** Laura A Bryant, Rossana Fanelli, Alexander J A Cobb

**Affiliations:** 1 School of Chemistry, Food and Pharmacy (SCFP), University of Reading, Whiteknights, Reading, Berks RG6 6AD, United Kingdom

**Keywords:** bifunctional, cupreidine, cinchona, cupreine, organocatalysis

## Abstract

Cinchona alkaloids with a free 6'-OH functionality are being increasingly used within asymmetric organocatalysis. This fascinating class of bifunctional catalyst offers a genuine alternative to the more commonly used thiourea systems and because of the different spacing between the functional groups, can control enantioselectivity where other organocatalysts have failed. In the main, this review covers the highlights from the last five years and attempts to show the diversity of reactions that these systems can control. It is hoped that chemists developing asymmetric methodologies will see the value in adding these easily accessible, but underused organocatalysts to their screens.

## Introduction

**Figure 1:**
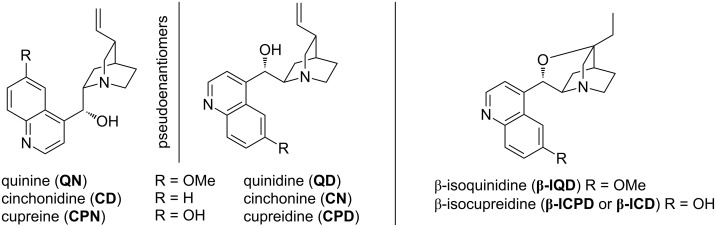
The structural diversity of the cinchona alkaloids, along with cupreine, cupreidine, β-isoquinidine and β-isocupreidine derivatives.

The cinchona alkaloids, comprising quinine (**QN**), quinidine (**QD**), cinchonidine (**CD**), cinchonine (**CN**, Figure 1), and their derivatives have revolutionized asymmetric catalysis owing to their privileged structures. The functional groups within these catalysts are highly pre-organized [1–2] and can both coordinate to, and activate the components of a reaction in a well-defined manner, thus facilitating a stereocontrolled process. The ability to easily derivatise these catalyst systems in a bespoke fashion in order to optimize their stereoselective behaviour has seen their utility burgeon dramatically over the last decade. Of particular note is the use of these cinchona systems within bifunctional thiourea catalysis [3–12].

Cupreine (**CPN**) and cupreidine (**CPD**), the non-natural demethylated structures of quinine and quinidine, respectively, have also found extensive utility, but not to the same extent, which is surprising given the broad range of chemistries that they have been shown to facilitate, and which are the subject of this review.

Herein, we describe the highlights of **CPN**, **CPD** and their derivatives in asymmetric organocatalysis over the last five years or so [13–14]. The review is organized by reaction type, beginning with the Morita–Baylis–Hillman process – one of the first reactions to utilize 6’-OH-cinchona alkaloid derivatives in asymmetric organocatalysis. The focus will then turn to asymmetric 1,2-additions followed by conjugate additions, a cyclopropanation, (ep)oxidations, α-functionalisation processes, cycloadditions, domino processes and finally miscellaneous reactions. We ultimately aim to demonstrate through this plethora of diverse processes, that the 6’-OH cinchona class of alkaloids are a dynamic and versatile type of organocatalyst that should be included in the screening libraries of chemists seeking to develop asymmetric methodologies.

## Review

### Morita–Baylis–Hillman (MBH) and MBH-carbonate reactions

**Scheme 1:**
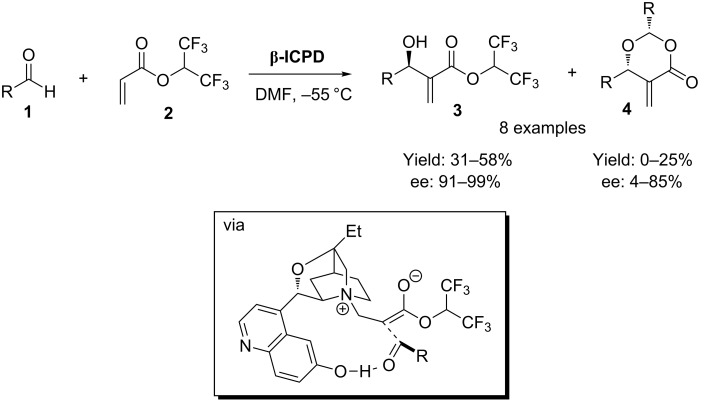
The original 6’-OH cinchona alkaloid organocatalytic MBH process, showing how the free 6’-OH is essential for coordination to the substrate.

The first reports of an asymmetric reaction catalyzed by a cinchona organocatalyst with a 6’-OH functionality came from Hatakeyama and co-workers in 1999 who demonstrated the use of **β-ICPD** in an asymmetric Morita–Baylis–Hillman (MBH) reaction [15–18] what is essentially an asymmetric C3-substituted ammonium enolate reaction (Scheme 1) [19–20]. In this classic process, it was hypothesized that the 6’-OH group was critical in directing the incoming aldehyde electrophile (see Scheme 1 box).

**Scheme 2:**
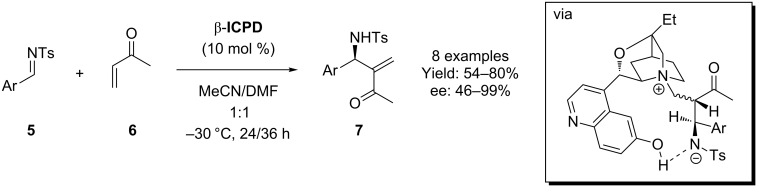
Use of **β-ICPD** in an aza-MBH reaction.

Soon after, Shi and co-worker demonstrated the use of **β-ICPD** in the reaction of imines **5** with methyl vinyl ketone (MVK, **6**) using the same catalyst (Scheme 2) [21]. The same study investigated methylacrylate and acrylonitrile as the conjugated partner, but these were less successful. Shi proposed a similar reaction mechanism for this process, whereby the 6’-OH functionality is critical in the control of stereoselectivity.

**Scheme 3:**
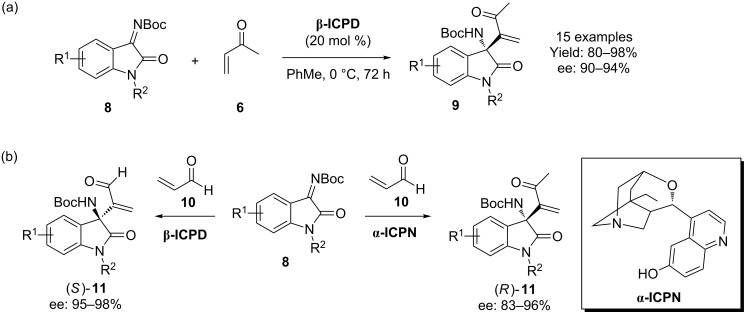
(a) The isatin motif is a common feature for MBH processes catalyzed by **β-ICPD**, as demonstrated by Shi and Li and co-workers. (b) Takizawa and co-workers demonstrated similar chemistry, but also utilized the catalyst **α-ICPN** (inset).

In a more recent extension of this work, Shi, Li and co-workers partnered the isatin derived *N*-Boc ketimines **8** with MVK (**6**, Scheme 3a) to obtain the corresponding adducts **9** with very good selectivity [22]. Interestingly, replacing the Boc group with an ethyl carbamate decreased the yield and enantioselectivity dramatically, as did having a substituent at the 4-position of the ketimine. In a related study, Takizawa and co-workers demonstrated that the quinine derived organocatalyst, **α-ICPN** [23] produced the enantiomeric product in a similar process using acrolein **10** as the conjugate partner (Scheme 3b) [24].

**Scheme 4:**
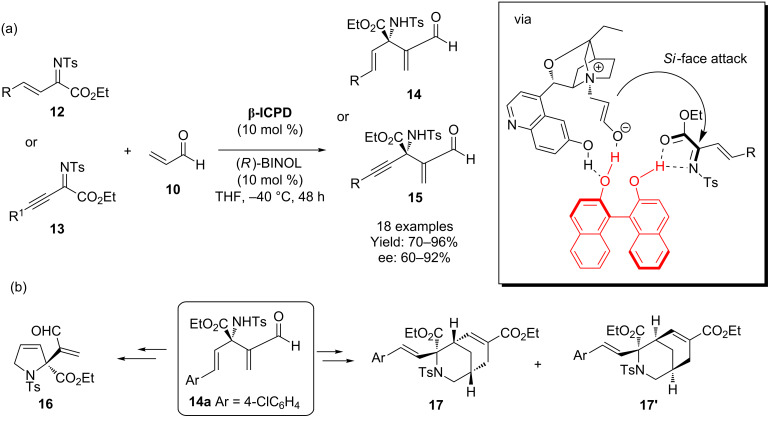
(a) Chen’s asymmetric MBH reaction. Good selectivity was dependent upon the presence of (*R*)-BINOL (shown in red) as well as **β-ICPD**. (b) Diverse structures were obtained from the MBH adduct **14a**.

Chen and co-workers developed an aza-MBH process using **β-ICPD** in the reaction between *N*-sulfonyl-1-aza-1,3-butadienes and activated alkenes (Scheme 4) [25]. In this report, optimal selectivity required (*R*)-BINOL as a co-catalyst (see inset for proposed catalytic transition state – (*R*)-BINOL shown in red). Furthermore, the utility of the adducts obtained was demonstrated through their conversion to a number of useful constructs (e.g., **16** and **17**).

**Scheme 5:**
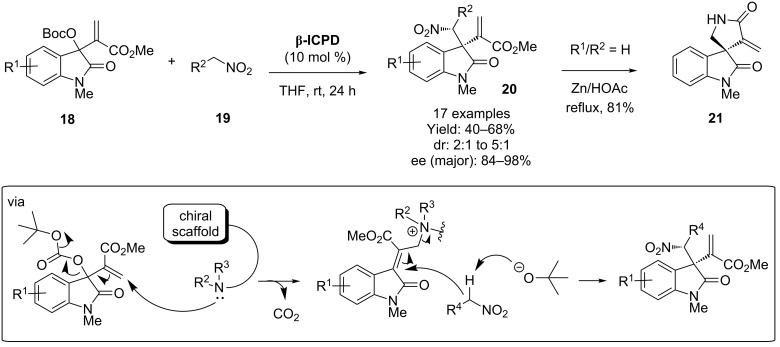
Lu and co-workers synthesis of a spiroxindole.

In reactions very much related to the MBH process, isatin derivatives have also proven to be particularly suited to the reaction of MBH-like products [26–28]. In these processes, the tertiary amine adds into the conjugate ester as with the MBH reaction, but instead of the resulting C3-ammonium enolate reacting with an electrophile, an E1cB elimination of the carbonate occurs to generate another conjugated system. This can then undergo an attack by a Michael donor; elimination of the catalyst then generates the *exo*-methylene adduct. For example, Lu and co-workers have used **β-ICPD** to react isatin-derived MBH carbonates **18** with nitroalkanes **19** [29]. The resulting adducts **20** could be converted to the corresponding spiroxindole **21** via a Zn/HOAc mediated reduction of the nitro functionality (Scheme 5).

**Scheme 6:**
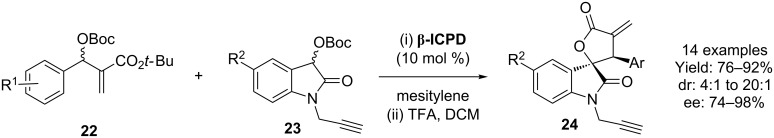
Kesavan and co-workers’ synthesis of spiroxindoles.

Similarly, Kesavan and co-workers reacted 3-*O*-Boc-oxindoles **23** with MBH carbonates **22** to generate a range of spirocyclic scaffolds containing α-*exo*-methylene-γ-butyrolactone **24** – again using **β-ICPD** (Scheme 6) [30].

### Nazarov cyclization

**Scheme 7:**
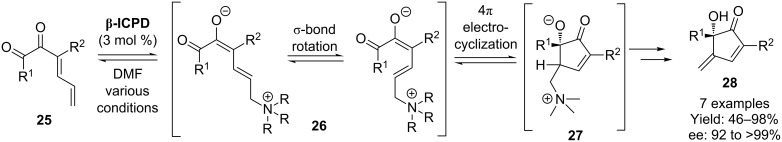
Frontier’s Nazarov cyclization catalyzed by **β-ICPD**.

An asymmetric Nazarov cyclization has been developed by Frontier and co-worker using **β-ICPD** through a mechanism that is reminiscent of the MBH reaction (Scheme 7) [31]. In this process however, the tertiary amine adds to the conjugated system **25** in a 1,6-fashion to generate intermediate enolate **26**. This undergoes a single bond rotation to set up a 4π-electrocyclization, generating second intermediate **27**. Elimination of the tertiary amine then gives γ-methylene cyclopentenone **28**.

### 1,2-Addition reactions

#### Henry reaction

**Scheme 8:**
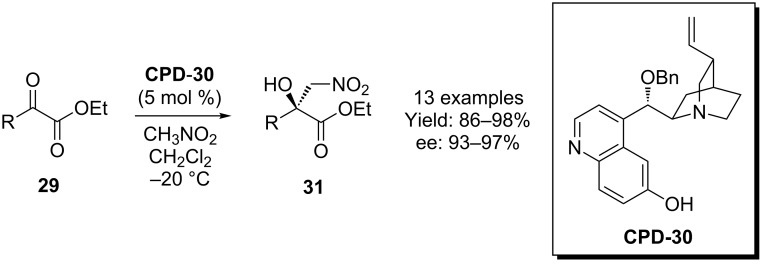
The first asymmetric nitroaldol process catalyzed by a 6’-OH cinchona alkaloid.

The use of cupreine and cupreidine derivatives in the addition of nitroalkanes to carbonyl compounds was first demonstrated by Deng and co-workers [32–34]. In this excellent study, catalysts substituted with benzyl at the 9-OH position gave the best results (**CPD-30**, Scheme 8). This report also demonstrated that the enantiomer of β-nitroester **31** could be obtained using the corresponding pseudoenantiomeric organocatalyst with comparable results.

**Scheme 9:**
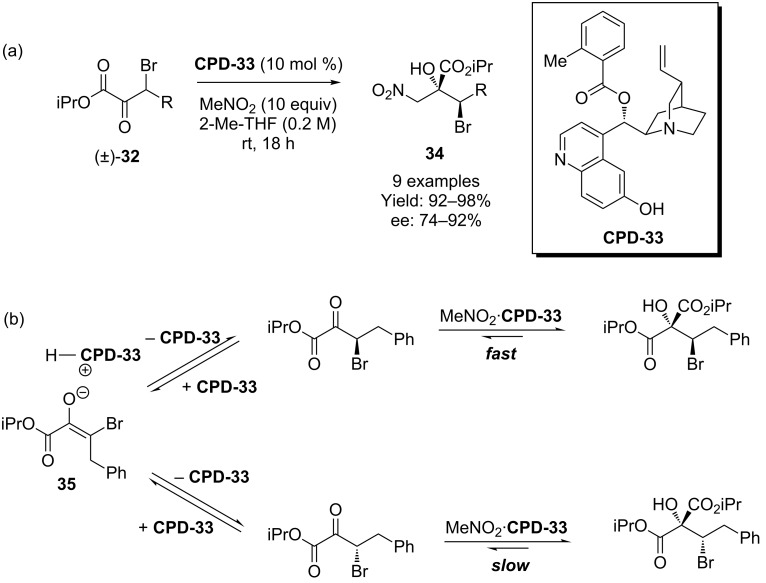
A cupreidine derived catalyst induces a dynamic kinetic asymmetric transformation.

More recently, Johnson and co-worker used the *o*-toluoyl derived organocatalyst **CPD-33** to effect a dynamic kinetic asymmetric transformation of racemic β-bromo-α-keto esters **32** (Scheme 9a) [35]. The mechanism, deduced from deuterium labeling studies, proposes that one of the two enantiomers of **32** will react more rapidly with nitromethane in the presence of the cupreidine catalyst **CPD-33**. As these enantiomers equilibrate via **35** in the presence of the catalyst, a dynamic kinetic asymmetric reaction occurs (Scheme 9b).

#### Friedel–Crafts reaction

**Scheme 10:**
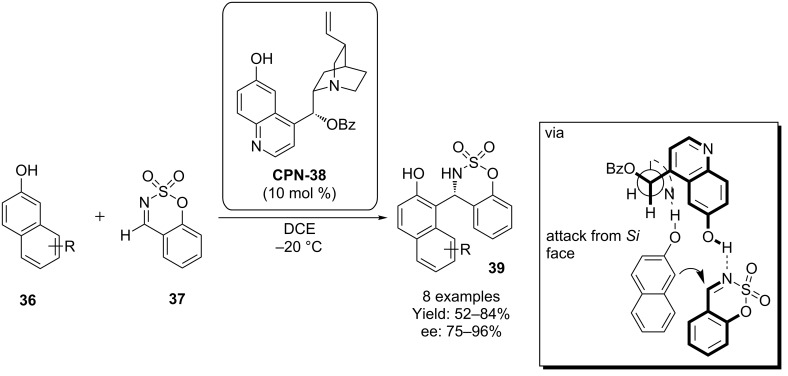
Cupreine derivative **38** has been used in an organocatalytic asymmetric Friedel–Crafts reaction.

Pedro and co-workers have utilized a 9-OH benzoyl derivatised cupreine **CPN-38** to effect a Friedel-Crafts reaction of 2-naphthols **36** with benzoxathiazine 2,2-dioxides **37** (Scheme 10). These cyclic imides, derived from salicylic aldehydes, have a rigid structure which prevents *E*/*Z*-isomerization, allowing for greater control over the stereochemical outcome of the reaction [36]. This work was based on a related scheme from Chimni and co-worker, who used **CPN** derivatised at the 9-OH with 1-naphthoyl in the addition of sesamol to a range of *N*-sulfonylimines [37].

### 1,4-Conjugate additions

**Scheme 11:**
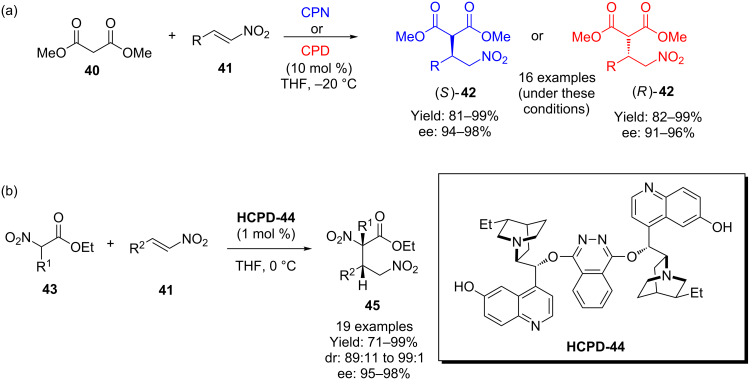
Examples of 6’-OH cinchona alkaloid catalyzed processes include: (a) Deng’s addition of dimethyl malonate into nitroolefins, and (b) Lin’s similar process with α-nitroesters using the symmetric hydrocupreidine system **HCPD-44**.

Deng and co-workers have contributed many examples of 1,4-additions that have been facilitated by **CPD** and **CPN** derived catalysts. For example, and amongst the earliest examples in the field, underivatized **CPD** or **CPN** were used in the addition of dimethyl malonate (**40**) to a range of nitrostyrenes **41**, giving the resulting adducts with excellent enantioselectivity (Scheme 11a) [38]. Subsequent reports by Deng and co-workers include, amongst many varieties of Michael acceptor and donor with various **CPN**/**CPD** derivatives [39–41], an example where β-ketoesters are used as the nucleophilic component [42]. It is on the basis of this work that Lin and co-workers were recently inspired to use the (*de*-Me-DHQ)_2_PHAL catalyst **HCPD-44** in the addition of α-substituted nitro acetates **43** also into nitroolefins **41** (Scheme 11b) [43–46].

**Scheme 12:**
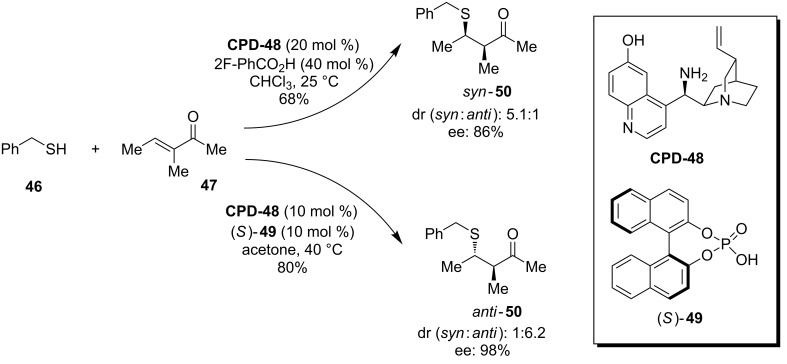
A diastereodivergent sulfa-Michael addition developed by Melchiorre and co-workers.

In a fascinating report, Melchiorre and co-workers use the 9-amino-CPD system **CPD-48** to control the Michael addition of thiols **46** into α-branched enones **47** via iminium ion catalysis (Scheme 12) [47]. This study found that the catalytic function could be modulated to induce diastereodivergent pathways by applying an external chemical stimulus (Scheme 12). Several conclusions were made from this study, one of which was that the hydrogen-bonding moiety of the 6’-OH in the catalyst is essential in directing the reaction towards the *anti*-diastereoselective pathway. Secondly, the solvent was critical in the diastereocontrol of the reaction. This is put down to the fact that the solvent can have an important influence on the conformation of the flexible cinchona framework, which has a knock-on effect on the catalytic outcome. Interestingly, the chiral nature of the binol phosphoric acid catalyst (*S*)-**49** in the *anti*-selective process was not thought to be hugely influential upon the stereochemical outcome. Indeed replacing it with diphenyl hydrogen phosphate (DPP) gave comparable results, ultimately leading to a third conclusion – that the strong hydrogen-bonding ability of the phosphate anion will favour the *anti*-selective pathway.

**Scheme 13:**
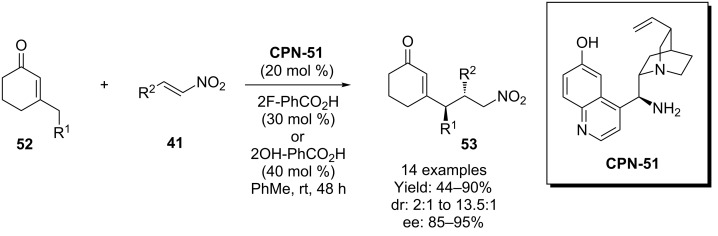
Melchiorre’s vinylogous Michael addition.

Melchiorre and co-workers have also succeeded in using the related cupreine organocatalyst **CPN-51** in a direct vinylogous Michael addition reaction [48]. In this process, cyclic enones **52** are added to nitroalkenes **41** using dienamine catalysis (Scheme 13). Although no model is suggested with respect to how the 6’-OH is involved, it is clearly of importance as the analogous 6’-OMe derived cupreine catalyst gives significantly lower conversions and selectivities.

**Scheme 14:**
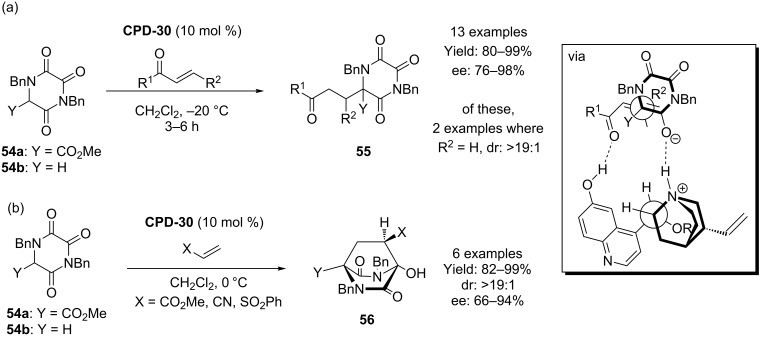
Simpkins’s TKP conjugate addition reactions.

Simpkins and co-workers have used **CPD-30** in the reaction of triketopiperidines (TKPs) **54** with a variety of enones with very good selectivity (Scheme 14a) [49]. Interestingly, with different types of acceptor, a cyclization event occurred leading to the bicyclic hydroxydiketopiperizine system **56** with very high diasterecontrol. Once again, the authors invoke a critical role for the 6’-OH group in the co-ordination and activation of the electrophile in these processes.

### Cyclopropanations

**Scheme 15:**
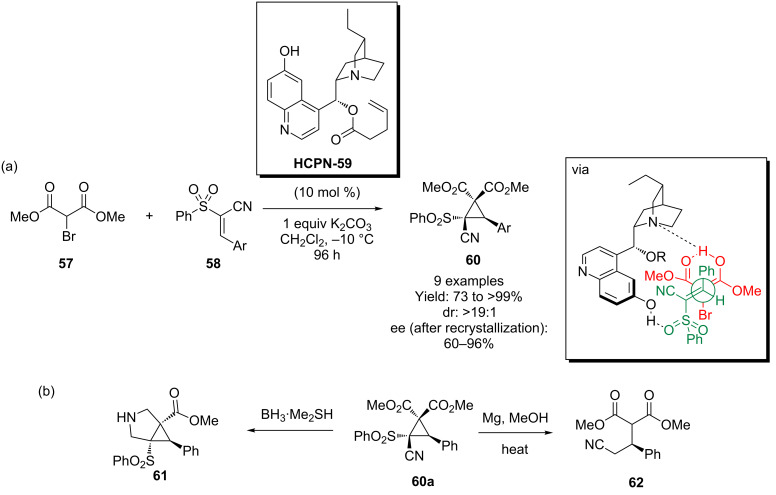
Hydrocupreine catalyst **HCPN-59** can be used in an asymmetric cyclopropanation.

Not unrelated to the Michael addition in a mechanistic sense, is the asymmetric cyclopropanation using dimethyl bromomalonate (**57**) and some form of Michael acceptor. In this process, the enolate resulting from the initital conjugate addition attacks the C–Br bond to form a three-membered ring. In our work in this area, we designed a new cupreine derived catalyst **HCPN-59** to add dimethyl bromomalonate (**57**) to a conjugated cyanosulfone **58** (Scheme 15). Our expectations were that the highly functionalized adduct **60** that resulted would be able to undergo a variety of chemistries, allowing access to a number of diverse scaffolds. This was demonstrated through the synthesis of the corresponding 3-azabicyclo[3.1.0]hexane system **61** and the δ^3^-amino acid precursor **62** [50].

### Epoxidations and oxaziridinations

**Scheme 16:**
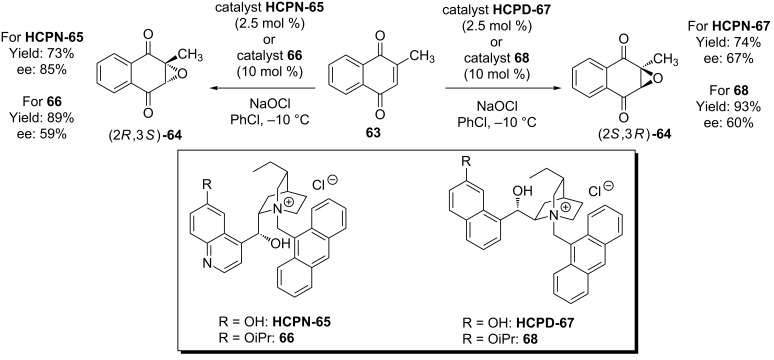
The hydrocupreine and hydrocupreidine-based catalysts **HCPN-65** and **HCPD-67** demonstrate the potential for phase transfer catalyst derivatives of the 6’-OH cinchona alkaloids to be used in asymmetric synthesis.

A variety of cinchona-derived phase transfer catalysts have been employed in the asymmetric epoxidation [51–52], but only one utilizes the free 6’-OH. In this report by Berkessel and co-workers, cupreine and cupreidine PTCs **HCPN-65** and **HCPD-67** were used in the epoxidation of the *cis*-α,β-unsaturated ketone **63** with sodium hypochlorite [53–54]. Interestingly, the use of these pseudoenantiomers did not lead to similar magnitudes of stereoselection in the opposite enantiomers of epoxide **64** that they produced, as is often the case with cinchona alkaloid catalyzed processes. However, the role of the 6’-OH was clearly important when directly compared with the equivalent 6’-OiPr catalysts **66** and **68** (Scheme 16).

**Scheme 17:**
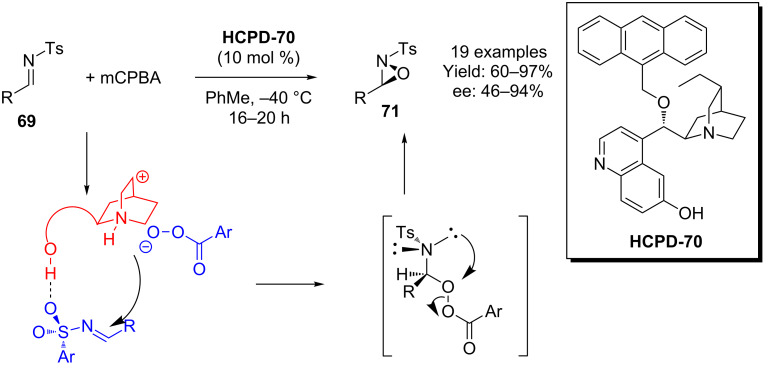
Jørgensen’s oxaziridination.

Jørgensen and co-workers have used another anthracenyl-modified hydrocupreidine **HCPD-70** in an enantioselective oxaziridination using mCPBA as the oxidant (Scheme 17) [55]. The authors propose that the quinuclidine nitrogen is protonated by the peracid, giving rise to a tight ion pair, whilst the 6’-OH coordinates to the sulfonyl group oxygen, thus bringing the reactants together. Subsequent reaction then leads to an intermediate α-aminoperoxy structure, which quickly collapses to the oxaziridine **71**.

### Formation of C–X bonds α-functionalisation

**Scheme 18:**
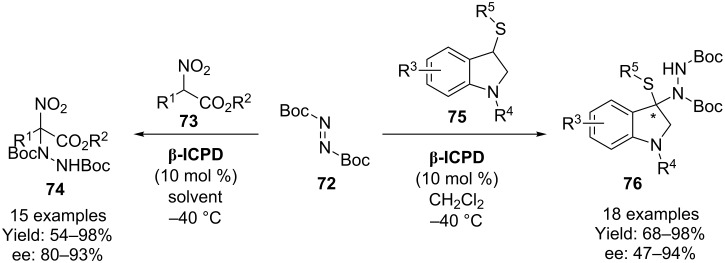
Zhou’s α-amination using **β-ICPD**.

In two separate reports, Zhou and co-workers demonstrate the use of di-*tert*-butyl azodicarboxylate **72** (DBAD) in the direct amination of several different substrates using β-isocupreidine (**β-ICPD**). In the first of these, α-substituted nitoacetates **73** are used [56], and in the second 3-thiooxindoles **75** are employed (Scheme 18) [57]. Unfortunately, in neither of these papers is the absolute stereochemistry elucidated.

**Scheme 19:**
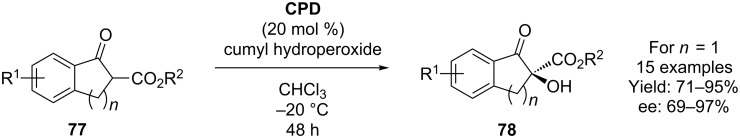
Meng’s cupreidine catalyzed α-hydroxylation.

Finally, Meng and co-workers used cupreidine (**CPD**) in the α-hydroxylation of indenones (where *n* = 1 in **77**) using cumyl hydroperoxide (Scheme 19) [58]. Interestingly, the 3,4-dihydronaphthalen-1(2*H*)-one derivative (where *n* = 2 in **77**) did not afford any detectable product.

### Transamination

**Scheme 20:**
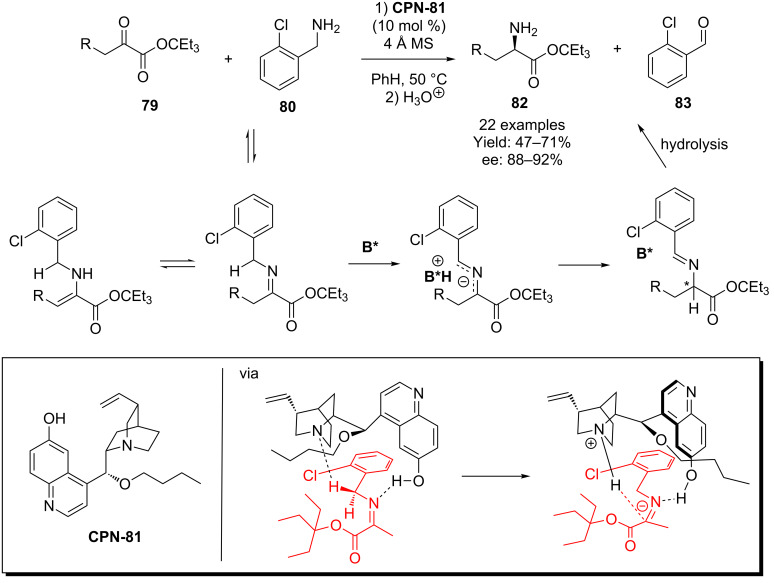
Shi’s biomimetic transamination process for the synthesis of α-amino acids.

A range of α-amino acid derivatives have been accessed by Shi and co-workers who developed an organocatalytic transamination process using the cupreine catalyst **CPN-81**, which is substituted with *n*-butyl at the 9-OH position [59]. In this report, the α-ketoester **79** was reacted with the primary amine *o*-ClC_6_H_4_CH_2_NH_2_ **80** in the presence of the catalyst. Once again, the role of the 6’-OH functionality is shown to be critical in the orchestration of the reaction process, as depicted in the proposed transition state model (Scheme 20).

### Cycloadditions

**Scheme 21:**
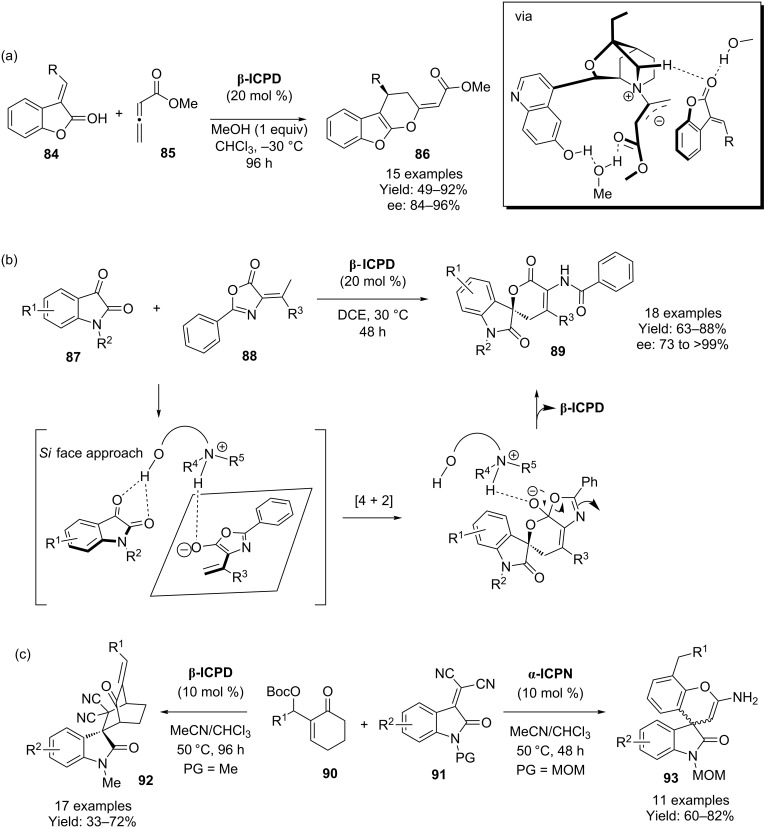
β-Isocupreidine catalyzed [4 + 2] cycloadditions.

The [4 + 2] cycloaddition of benzofuran-2(3*H*)-one derivatives **84** with methyl allenoate **85** to give the corresponding dihydropyran fused benzofuran precursors **86** using **β-ICPD** has been achieved by Li and Cheng and co-workers (Scheme 21a) [60–63]. A large number of computational studies were conducted to explain the enantioselection of the process, resulting in the transition state shown which depicts a critical methanol bridge, explaining the need for this as an additive within the reaction to give optimal stereoselectivities. Similarly, Xu and co-workers have used **β-ICPD** in the cycloaddition between isatin framework **87** and olefinic azlactones **88** to give adduct **89** (Scheme 21b) [64].

Finally, in a remarkable demonstration of diversity-oriented synthesis, Chen and co-workers have shown that simply by switching the type of 6’-OH cinchona-derived catalyst used, two different products can be obtained in their reaction between the 2-cyclohexenone MBH derivative **90** and isatylidene malontirile **91**, one of which is the [4 + 2] adduct **92**, albeit achieved in a step-wise manner (Scheme 21c) [65]. Disappointingly, though not uninteresting, is the fact that there is no enantioinduction for either of these processes.

**Scheme 22:**
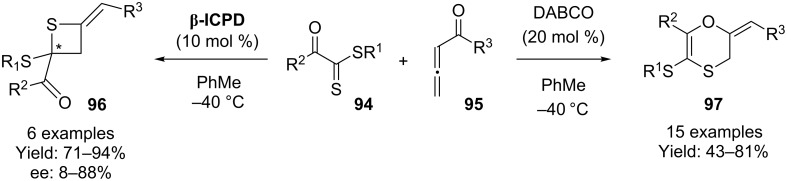
β-Isocupreidine catalyzed [2+2] cycloaddition.

β-Isocupredeine has also been used in the [2 + 2]-addition between 2-thioxoacetates **94** and allenoates **95** to give the corresponding thietanes **96**. In another example of how a different catalyst can lead to a different product, the authors demonstrated that the use of DABCO led instead to the [4 + 2] adduct (Scheme 22) [66].

### Domino reaction

**Scheme 23:**
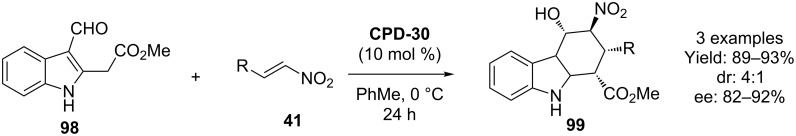
A domino reaction catalyst by cupreidine catalyst **CPD-30**.

Although it could be argued that some of the reactions within this review are already domino reactions (e.g., MBH, and the cyclopropanation), a recent and clearer example of the use of a 6’-OH cinchona derived catalyst in such a process comes from the laboratory of Samanta and co-workers [67–68]. They have demonstrated an enantioselective domino reaction between 3-formylindoles **98** and nitroolefins **41** to generate the corresponding tricyclic adducts **99** using cupreidine derivative **CPD-30** (Scheme 23). Although the substrate scope for the enantioselective reaction is limited, the diastereoselectivities are reasonable, and the enantioselectivities are excellent.

### Other processes

#### Asymmetric oxidative coupling

**Scheme 24:**
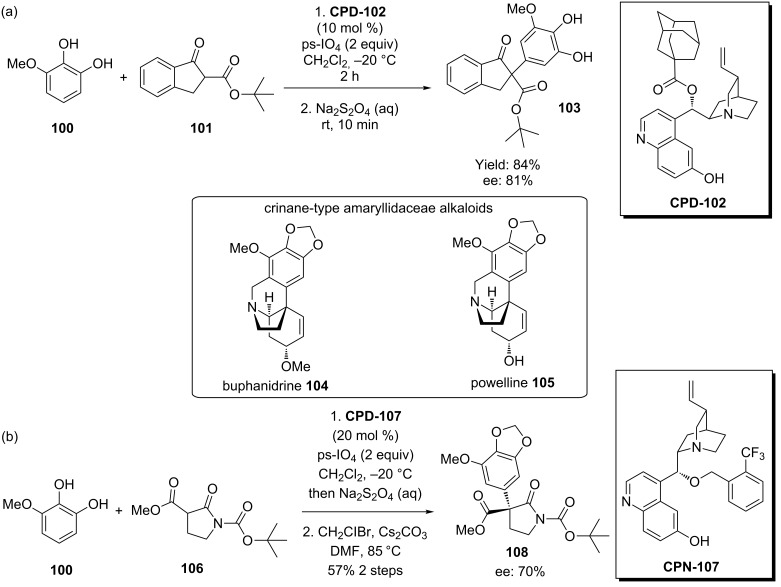
(a) Dixon’s 6’-OH cinchona alkaloid catalyzed oxidative coupling. (b) An asymmetric oxidative coupling en route to the attempted total synthesis of some amaryllidaceae alkaloids.

All carbon quaternary centers are prevalent in both natural and pharmaceutical compounds, but rank amongst the hardest to synthesize in a stereoselective manner. Dixon and co-workers have addressed this through the development of an asymmetric organocatalytic oxidative coupling – initially between 3-methoxycatechol (**100**) and *tert*-butyl 1-oxoindan-2-carboxylate (**101**) using an adamantane derivative of cupreidine **CPD-102** to give the corresponding adduct **103** in 84% yield and 81% ee (Scheme 24a) [69]. An attempt to develop this methodology towards an asymmetric total synthesis of buphanidrine (**104**) and powelline (**105**) led to the bespoke development of another cupreidine catalyst **CPN-107**. Unfortunately, although the resulting adduct **108** (after alkylation of the catechol) was produced in a 70% enantiomeric excess (Scheme 24b), subsequent steps that had worked with the racemic synthesis severely deteriorated this, preventing completion of the total synthesis [70–71].

## Conclusion

Cupreine and cupreidine and their derivatives have been demonstrated to be suited to a wide range of reaction processes, often with very good enantioinduction. In most cases these catalysts are easy to make from the corresponding cinchona alkaloids, making them attractive compounds for methodologists to have within their catalyst arsenal. They seem particularly suited to catalysis with systems that have an aromatic ring next to a five-membered ring – e.g., indoles, indenones, isatin etc*.* – especially when it comes to the Morita–Baylis–Hillman reaction, although they are in no way limited to these, and one can only expect the prevalence of these remarkable bifunctional catalysts within the literature to increase over the coming years.
